# Supplementary material for: Spatial structure of city population growth
Source: Nat Commun. 2022 Oct 8;13:5931. doi: 10.1038/s41467-022-33527-y (PMC9547901; doi:10.1038/s41467-022-33527-y)
Supplement: Supplementary file 1 — Supplementary Information [file 41467_2022_33527_MOESM1_ESM.pdf]

Supplementary Information: Spatial Structure of City Population Growth

Sandro M. Reia, P. Suresh C. Rao, Marc Barthelemy, Satish V. Ukkusuri[†]

Population growth and the role of domestic migration

ACS datasets (1) provide county-to-county flow files of 5-year period surveys from 2005-2009 to 2015-2019. Supplementary Fig. 1A, which shows the population and the number of people who changed address within the U.S. (viz. domestic migration) over the past 15 years, reveals that domestic migration is declining: about 46 million of people moved within the U.S. in the first survey (2005 - 2009) and approximately 43.5 million migrated within the country in the last one (2015 - 2019). The highest share of flows is within county (Supplementary Fig. 1B), followed by flows within state (but between different counties) and between states migration.

The growth of U.S. counties due to domestic migration can be seen in Supplementary Fig. 2, in which counties are colored according to the respective netflow (inflow - outflow) of people. The variety of colors highlight the heterogeneity of the netflows. We note that flows driven by COVID are not included in our analysis (3).

Our study focuses on intra- and inter-city flows to analyze the population growth of U.S. counties. Supplementary Fig. 3 shows that the distribution of x, defined as the ratio of the magnitudes of netflows and natural growth, has an average of ≈ 9, indicating that domestic netflows are about nine times more intense than natural growth. The cumulative distribution indicates that more than 76% of the counties present domestic migratory netflows more intense than natural growth. Supplementary Fig. 4 shows that domestic netflows are more intense than natural growth irrespective of the size of the county under consideration. In Supplementary Fig. 4A, the average of x is higher than 1 for all the intervals considered. Supplementary Fig. 4B shows that the probability of finding $x \geq 1$ is higher than 0.5 in all population intervals. The results presented in Supplementary Fig. 3 and Supplementary Fig. 4 stresses the relevance of our approach: by studying the effects of flows on the growth of U.S. cities at the county level, we are addressing the heterogeneity and the structure of the main process that drives city growth.

In order to convey a better picture of the spatial structure of population growth in the U.S. metro areas, in Supplementary Fig. 5A we show the relative population growth as function of the distance from the core county for the 46 metro areas with at least 5 counties. Relative population growth is defined as $(S(2019) - S(2015))/S(2015)$, in which $S(2015)$ and $S(2019)$ are the county population in 2015 and 2019, respectively. The correlation R and p value suggest no correlation between distance and relative population growth. Supplementary Fig. 5B presents the relative natural growth as function of the distance from the core county. Relative natural growth is defined as total births (from 2015 to 2019) minus total deaths (from 2015 to 2019) in a county adjusted by county population. There is a small negative trend with the distance, indicating that core counties have more relative natural growth than external ones.

The heterogeneity of intra-state flows is presented in Supplementary Fig. 6. Within state flows grow super-linearly with the state population, as suggested by the log-log regression in Supplementary Fig. 6A ($y = ax^b, a =$

[†] sukkusur@purdue.edu.

$3.04 \cdot 10^{-5}, b = 1.42, R^2 = 0.92$). Two major components of inter-state moves are intra-city and inter-city flows (Supplementary Fig. 6B). Given that our data have population flows at the county level, 149 MSAs composed of only one county do not have intra-city flows. There are two states that have only one MSA, Vermont and Rhode Island. For these states, inter-city flows are equal to zero.

Flows between metro and micro, and metro and non-statistical areas

Supplementary Fig. 7 shows metro and micro, and metro and non statitical areas migratory patterns for all cities with more than 5 counties. The percentage of inflows from micropolitan statistical areas presents a slightly positive correlation with the destination county distance from the core county of the city (Supplementary Fig. 7A). The same pattern is observed with respect to the percentage of inflows from non statistical areas (Supplementary Fig. 7B).

The percentage of outflows to micro areas (Supplementary Fig. 7C) and the percentage of outflows to non statistical areas (Supplementary Fig. 7D) present a slightly positive correlation with the origin county distance from the core county of the city. However, resulting neflows due to flows to and from micro areas (Supplementary Fig. 7E) and due to flows to and from non statistical areas (Supplementary Fig. 7F) indicate that inflows and outflows are roughly the same, thus the resulting in negligibly small contribution of metro and micro, and metro and non statistical area flows to the growth of cities

House prices and unemployment rate

A full explanation of the several socio-economic factors that might affect internal migration patterns in the U.S. is beyond the scope of this study, but here we present a brief overview of the factors commonly associated with intra-city flows, namely housing costs and employment rate. In Supplementary Fig. 8 we investigate the effects of house prices in driving intra-city netflows. The highest share of intra-city netflows of New York, Chicago and Washington are from counties with higher to lower house prices, as seen in Supplementary Fig. 8A. However, cities as Houston, Dallas and Philadelphia present a reverse trend, in which the majority of intra-city netflows are towards counties with higher housing prices. Supplementary Fig. 8B reveals the lack of a clear pattern of the fraction of intra-city netflows to counties with cheaper houses with city population size.

The role of unemployment rate in driving intra-city netflows is explored in Supplementary Fig. 9. Intra-city netflows of cities as Dallas and Philadelphia are mostly towards counties with lower unemployment rate (Supplementary Fig. 9A). Conversely, intra-city netflows of cities as Dallas, Houston and Atlanta are almost equally spread between counties with higher and lower unemployment rates. Nevertheless, $2/3$ of the 46 cities with at least 5 counties are dominated by intra-city netflows towards counties with lower unemployment rates.

Heterogeneity of international inflows

Inflows of international migrants accounts for about 4.6% of the migration in the U.S.. As seen in the main text, the amount of immigrants scale superlinearly with the city size, viz. $Y = Y_0 S^{\theta}$ in which $\theta = 1.19$ (95% CI $[1.13, 1.24]$), indicating that large cities have more inflows per capita of international migrants than small ones. With respect to the spatial distribution of international inflows within cities, Supplementary Fig. 10 shows

that international inflows are highly concentrated at the core counties of cities, and the share of international inflows decreases dramatically with the distance from the core county, thus external counties are barely affected by international immigrants.

Statistical structure of inter-city flows

The population growth of a city k within a system of cities can be written as:

$$\frac{\partial S_k}{\partial t} = \eta_k S_k + \sum_{j \in \mathcal{N}_k} (\mathfrak{J}_{j,k} - \mathfrak{J}_{k,j}), \tag{1}$$

in which S_k is the population of city k, η_k is a random variable Gaussian distributed accounting for out-of-system growth, $\mathcal{N}_k$ is the set of cities exchanging people with city k, and $\mathfrak{J}_{j,k}$ is the flow of people from city j to city k. Note that the second term on the right side of Supplementary Eq. (1) corresponds to the growth due to within-system netflows to city k.

In (4), Verbavatz and Barthelemy show that within-system netflows can be rewritten in terms of ζ_k^*, in which ζ_k^* is a function of the total netflows adjusted by population size. In this context, the growth equation of cities becomes

$$\frac{\partial S_k}{\partial t} = \eta_k S_k + D S_k^{\beta} \zeta_k^*, \tag{2}$$

where D is a constant and β is the exponent of S_k. Verbavatz and Barthelemy find that ζ_k^* is a random variable well approximated by a heavy-tailed distribution, viz. Lévy distribution, indicating that extreme migratory events are crucial in shaping the growth of cities.

Here, were are going to follow (4) to show that the distribution of reescaled netflows at the county level is bounded by an exponetial distribution, suggesting that counties are not affected by migratory shocks as cities. We assume that the flows $J_{i,k}$ between county i and metro area k (aggregated flows to and from all the counties belonging to MSA k) can be written as $J_{i,k} = I_0 S_i^{\mu} S_k^{\nu} x_{i,k}$, where I_0 is a constant, S_i is the population of county i, S_k is the total population of city k, $x_{i,k}$ accounts for random noises and high order effects, and μ and ν are the exponents of S_i and S_k, respectively. In order to reduce the number of free parameters in the expression for $J_{i,k}$, we define $I_{i,k} = J_{i,k}/S_i$, and in Supplementary Fig. 11A we show that $I_{k,i}/I_{i,k} = (S_i/S_k)^{\nu-\mu+1}$ can be written as a linear function of S_i/S_k, meaning that $\nu = \mu$. This result allows us to write $I_{i,k} = I_0 S_i^{\nu} S_k^{\nu-1} x_{i,k}$. Fitting the flow per capita $I_{i,k}$ versus $S_i^{\nu} S_k^{\nu-1}$ gives us $\nu = 0.34$ (Supplementary Fig. 11B).

In a second step, in order to analyze the occurrence of extreme events, we investigate how the fluctuations (described by the random variables $x_{i,k}$) affect the flows. Once we have found the form of $J_{i,k}$, the relative magnitude of the fluctuations can be estimated with the quantity $X_{i,k} = (J_{i,k} - J_{k,i})/I_0 S_i^{\nu}$. The sum of all rescaled netflows affecting the growth of a county i is then captured by $\zeta_i = (1/N_i) \sum_{k \in N_i} X_{i,k}$, in which N_i is the set of metro areas exchanging people with county i. The result of this analysis is presented in the main text.

Trends over different time periods

In this section, we investigate the intra- and inter-city trends over two additional time periods, namely 2005-2009 and 2010-2014. The results regarding 2005-2009 is presented in Supplementary Fig. 12. Panel A shows the

percentage of intra-city inflows as function of the distance to the core county. Intra-city inflows correspond to only ~ 0.3 of the inflows to the core county, while inter-city inflows correspond to about 0.6 (panel B). As the distance from the core county increases, the contribution of inter-city inflows to total inflows decrease, as observed for the period analyzed in the paper.

Similar patterns are observed when we analyze the percentage of outflows due to intra- and inter-city outflows (panels C and D). Again, intra-city outflows from the core county correspond to ~ 0.35 of the total outflows, while inter-city outflows is accounts for more than 0.5. As the distance from the core county increases, intra- and inter-city outflows slightly decrease since outflows to micro and non statistical areas areas slightly increase (similarly to Supplementary Fig. 7 for 2015-2019 period).

Panels E and D reveal the contribution of intra- and inter-city netflows to county population growth. Intra-city netflows are negative at the core county, and increase as the distance from the core county increases. This result suggests that there is a trend of intra-city netflows from central to external regions of metro areas. On the other hand, inter-city netflows are almost zero for most of the counties, and decrease below zero at the most external counties. These findings indicate that intra-city netflows play a major role in the population growth of the outer regions of cities. Similar patterns are observed for the 2010-2014 (Supplementary Fig. 13), but population gain due to intra-city netflows at external counties were more modest.

In the manuscript we reported that the highest share of intra-city netflows were directed towards lower population density counties for the period 2015-2019. In Supplementary Figs. 14 and 15, we observe that for both periods, viz. 2005-2009 and 2010-2014, intra-city netflows were mainly directed to lower density counties, thus presenting the same trend as reported in the manuscript.

Validation with IRS dataset

The robustness of our findings is here addressed with the usage of additional datasets from the Internal Revenue Service (IRS). IRS releases annual county to county migration flow data based on the changes of addresses reported on filled taxes. Supplementary Figs. 16, 17, 18, 19 show percentage of inflows, outflows, and netflows adjusted by county population as function of the distance from core county for periods 2015-2016, 2016-2017, 2017-2018, 2018-2019, respectively, covering the ACS period reported in the paper (from 2015 to 2019).

In all cases, intra-city inflows and outflows are more frequent at external counties than inter-city inflows and outflows, respectively. However, IRS data indicate that intra-city flows account for more than 70% of inflows and outflows. A word is in order about the difference between both datasets. ACS data report about 18 million county-to-county domestic flows per year, while IRS data report about 10 million county-to-county domestic flows per year. ACS estimates flows based on statistical samples of the entire population, while IRS report flows based on the number of filled taxes. Besides, due to privacy reasons IRS suppress flows between counties in which the number of filled taxes is lower than 20. Given that many individuals are not required to file individual tax return because they make less than a certain amount for the corresponding year, IRS data might not fully capture the migration patterns of the entire U.S. population since the data does not include the ones below an income threshold.

Differences aside, ACS and IRS datasets lead to the same conclusion: Panels E and D of Supplementary Figs. 16, 17, 18, 19 reveal that intra-city neflows is negative at the core county, and becomes positive and increases as distance from core county increases, thus corroborating the finding that intra-city netflows are directed towards external counties. Given that inter-city netflows are about zero irrespective of the distance to core county, intra-city netflows play a major role in the population growth of the external regions of cities. As we can see, the results obtained with the IRS data supports the main conclusions drawn in the manuscript with the usage of data from ACS.

References

[1] County-to-County Migration Flows. https://www.census.gov/topics/population/migration/guidance/county-to-county-migration-flows.html, Accessed: 2022-01-10.

[2] Riordan Frost. Are Americans Stuck in Place? Declining Residential Mobility in the U. S., 2020.

[3] Xiaorong Jiang, Wei Wei, Shenglan Wang, Tao Zhang, and Chengpeng Lu. Effects of covid-19 on urban population flow in china. *International Journal of Environmental Research and Public Health*, 18(4):1617, 2021.

[4] Vincent Verbavatz and Marc Barthelemy. The growth equation of cities. *Nature*, 587(7834):397–401, 2020.

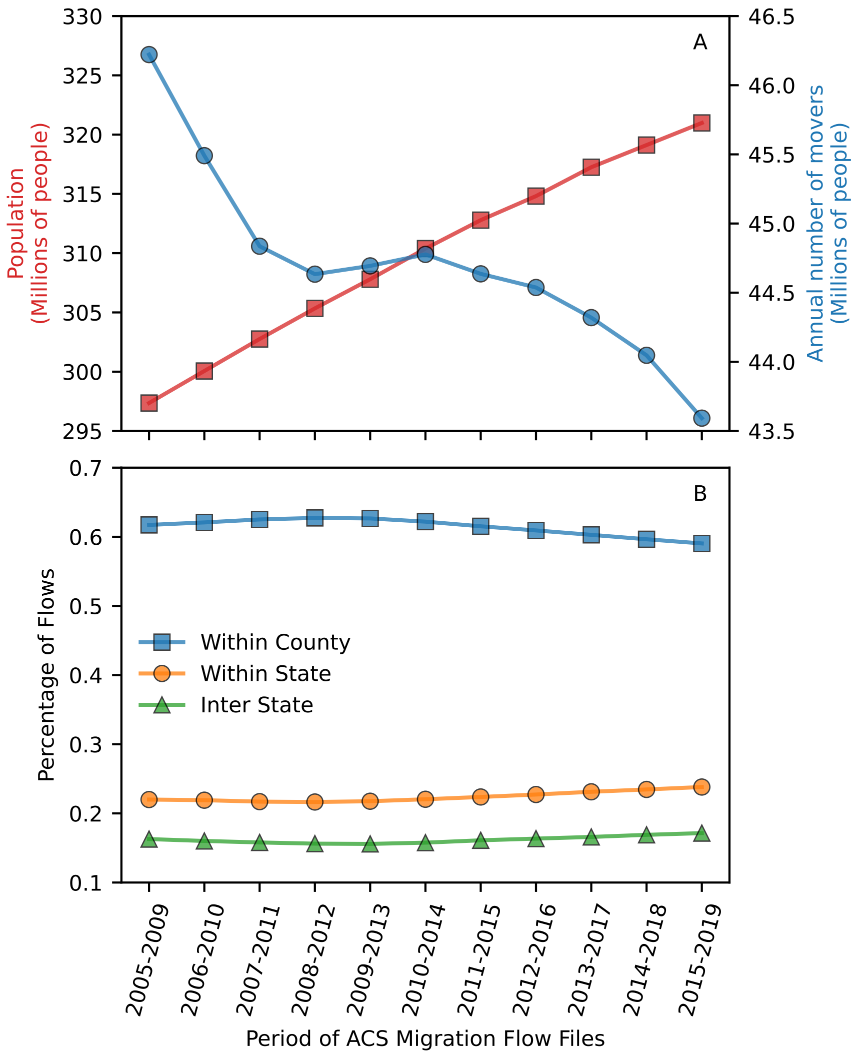

Supplementary Fig. 1: U.S. population and number of movers within the U.S. over the years. The U.S. population has been increasing from the first American Community Survey $(2005-2009)$ to the last one $(2015-2019)$ considered in our study, while the annual number of movers within the U.S. in each period has decreased (A). The percentage of flows (B) reveals that domestic flows within county has slightly decreased while domestic flows within and inter state have slightly increased from the $2008-2012$ period onward. The highest share of flows is within county, irrespective of the time period considered (2).

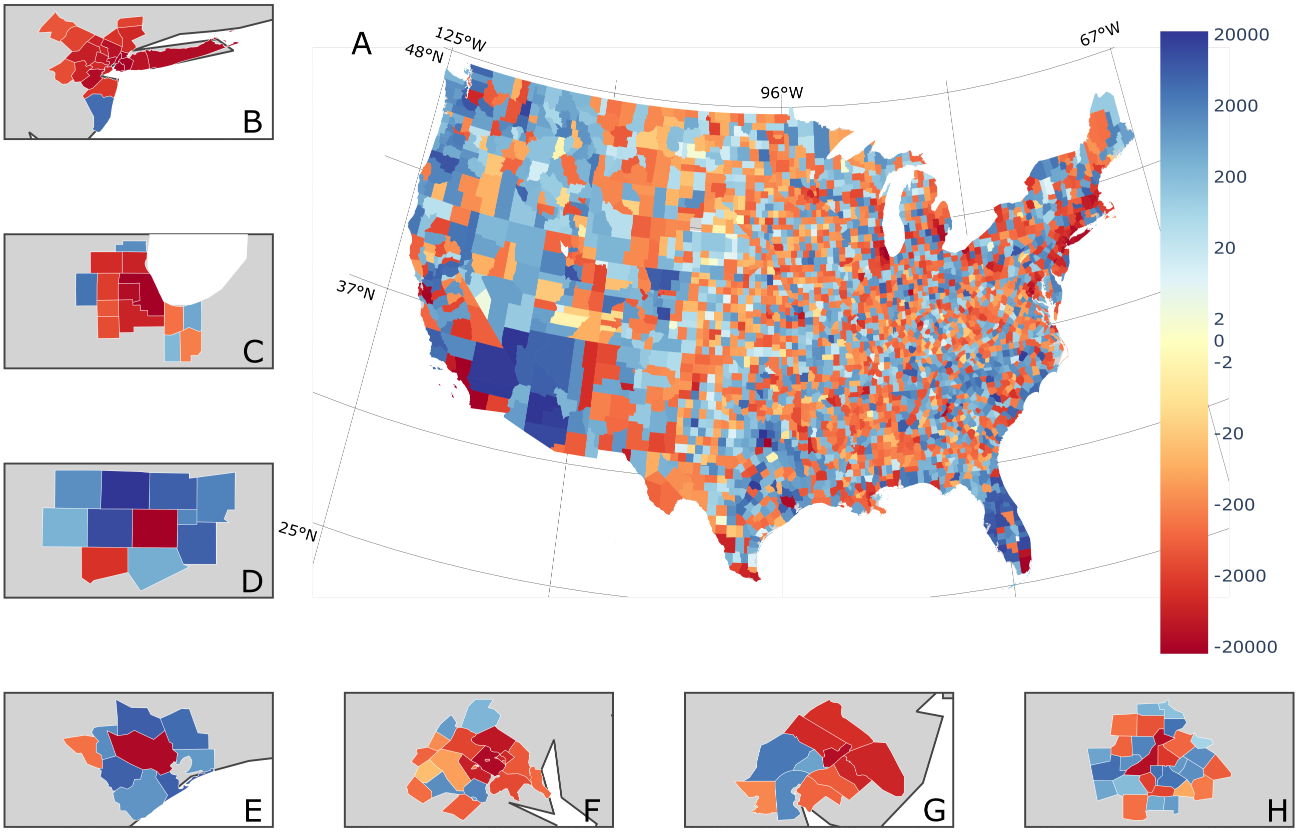

Supplementary Fig. 2: Spatial heterogeneity of domestic netflows of the U.S. counties. The map (A) shows the resulting netflow (inflow minus outflow) of people due to county-to-county annual relocations from 2015 to 2019. There is a large number of counties with positive netflow (blue) in the south and the west, while the counties with negative netflows (red) are localized in the north-central and north-east regions of the country. The color bar on the right shows the magnitude of netflows. Alaska and Hawaii are not shown. Panels (B-H) display a close-up of the counties belonging to the 7 most populous Metropolitan Statistical Areas with more than 5 counties, and which are: New York (B), Chicago (C), Dallas(D), Houston (E), Washington D.C. (F), Philadelphia (G), Atlanta (H). We observe that the netflow of core counties is negative and that external counties are more attractive (during 2015-2019 period) with positive annual netflows.

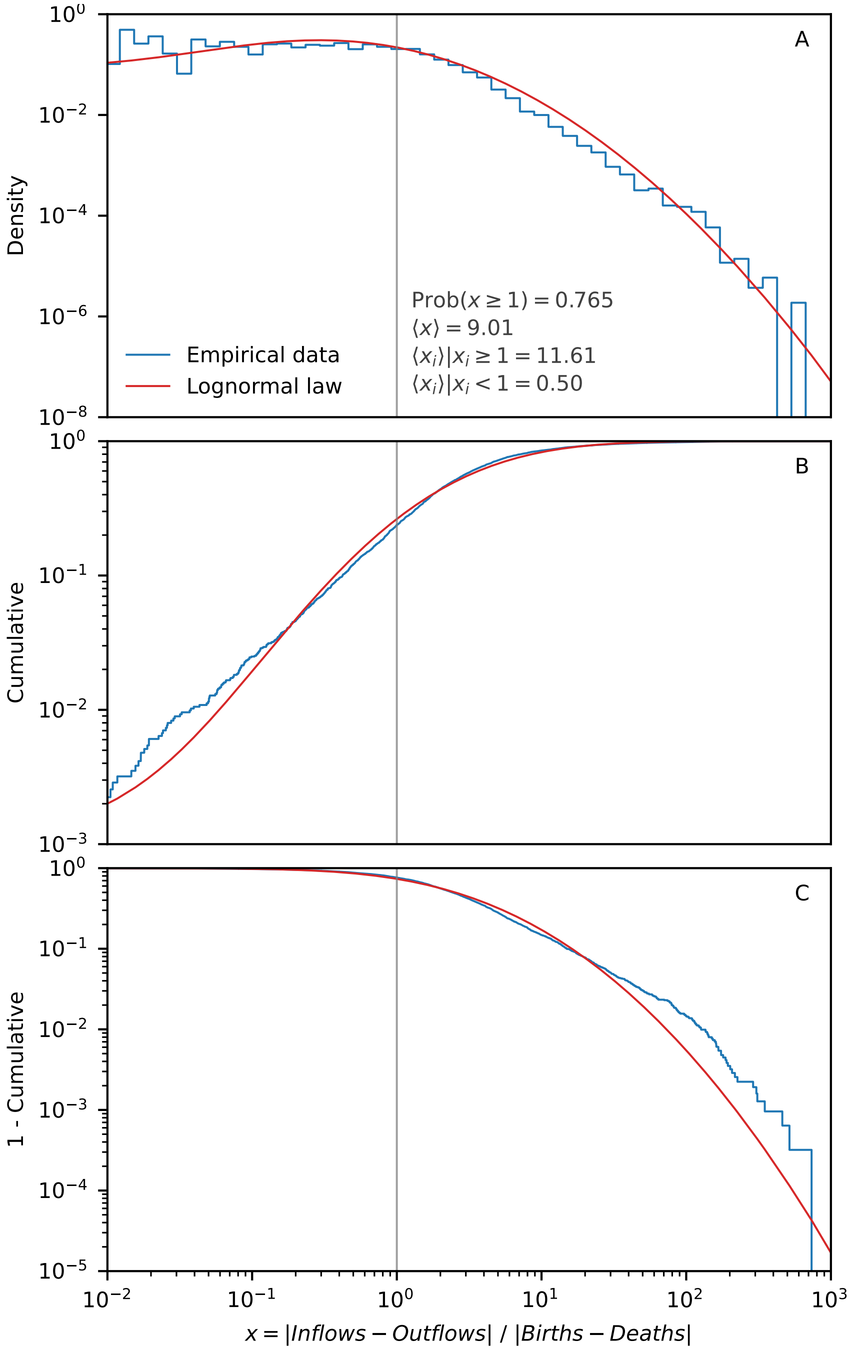

Supplementary Fig. 3: Domestic netflows are more intense than natural growth. The average of the distribution of x reveals that netflows from domestic migration are about nine times more intense than natural growth, and more than 76% of the 3141 counties have domestic netflows more intense than natural growth.

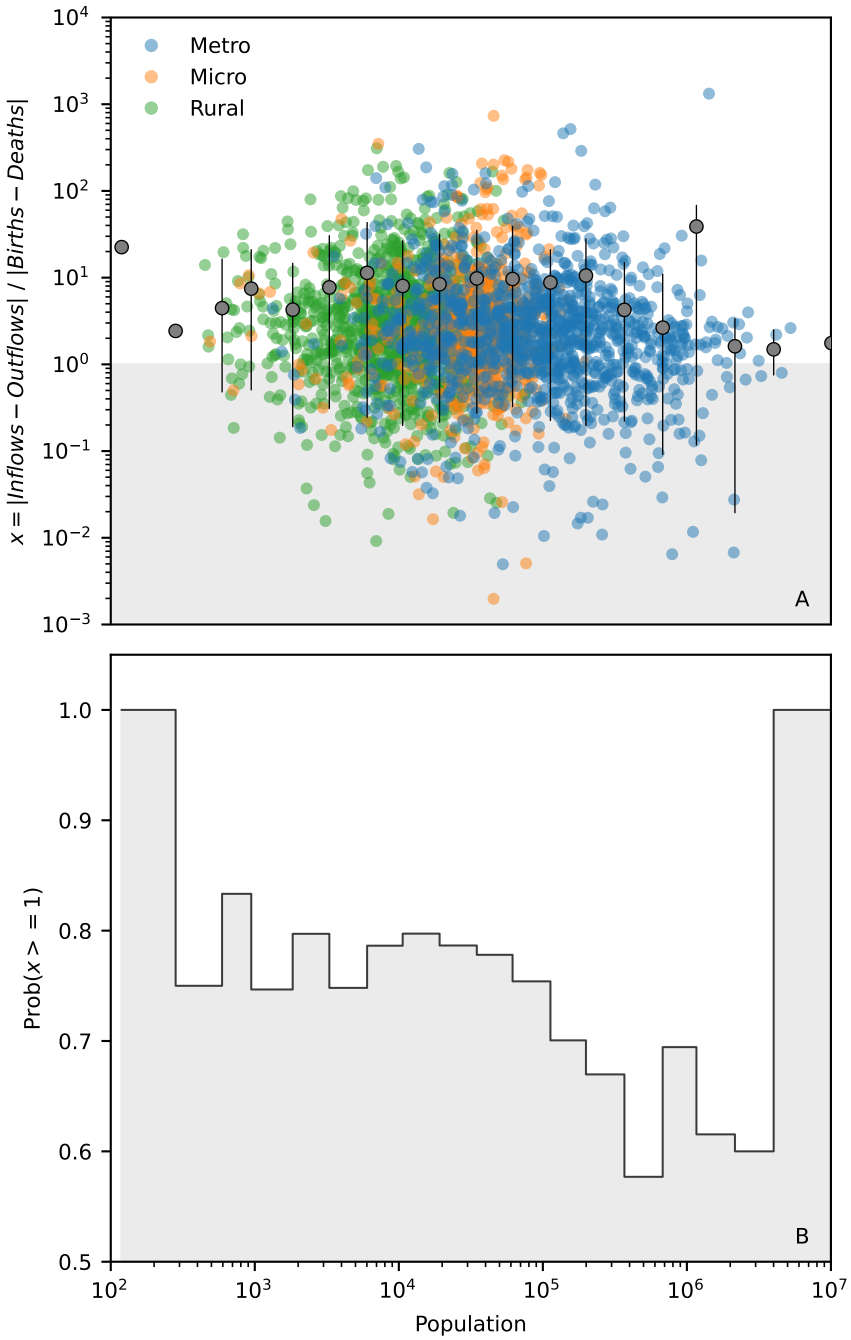

Supplementary Fig. 4: Netflows from domestic migration are more intense than natural growth irrespective of the county population. Panel A shows the average of x (gray dots) for different population intervals. The range of population is split into equally log-spaced bins. The number of counties n within each bin, from left to right, is 1, 1, 12, 30, 75, 138, 266, 454, 587, 534, 365, 248, 177, 109, 78, 36, 13, 5, 1. The black dots and the error bars indicate the mean and the 90% interval, respectively, of the counties within the corresponding bin. Panel B shows the probability of finding $x \geq 1$ for the same intervals of panel A. Note that $\langle x \rangle \geq 1$ and $P(x \geq 1)$ for all the intervals considered. Interestingly, x approaches one for the largest cities (population $> 10^6$).

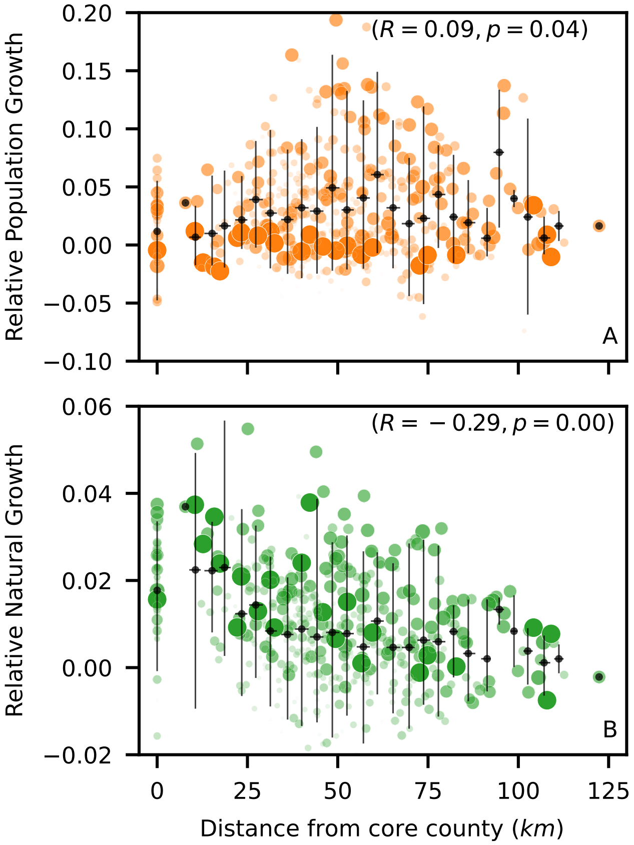

Supplementary Fig. 5: Relative population growth is approximately uniform over the different counties within a city, while natural growth is is stronger at the core counties. We define the core county as the one with the highest population density, and we plot in panel A the relative population growth (viz. $(S(2019) - S(2015))/S(2015)$) of each county within a city as a function of its distance to the core county. Panel B shows the relative natural growth (viz. $(Births(2015 - 2019) - Deaths(2015 - 2019))/S(2015)$) as function of the core county. In each panel, we indicate the Pearson correlation coefficient R and the associated p value. The sizes of red circles and blue squares are proportional to the city population. The range of distances is split into equally spaced bins. The number of counties n within each bin, from left to right, is 46, 1, 4, 7, 7, 17, 21, 31, 36, 38, 34, 31, 31, 30, 20, 20, 21, 14, 17, 9, 9, 6, 4, 2, 5, 5, 2, 1. The black dots and the error bars indicate the mean and the 90% interval, respectively, of the counties within the corresponding bin. We also show the Pearson correlation coefficient R and the p-value associated with the two-sided test of the null hypothesis of non-correlation.

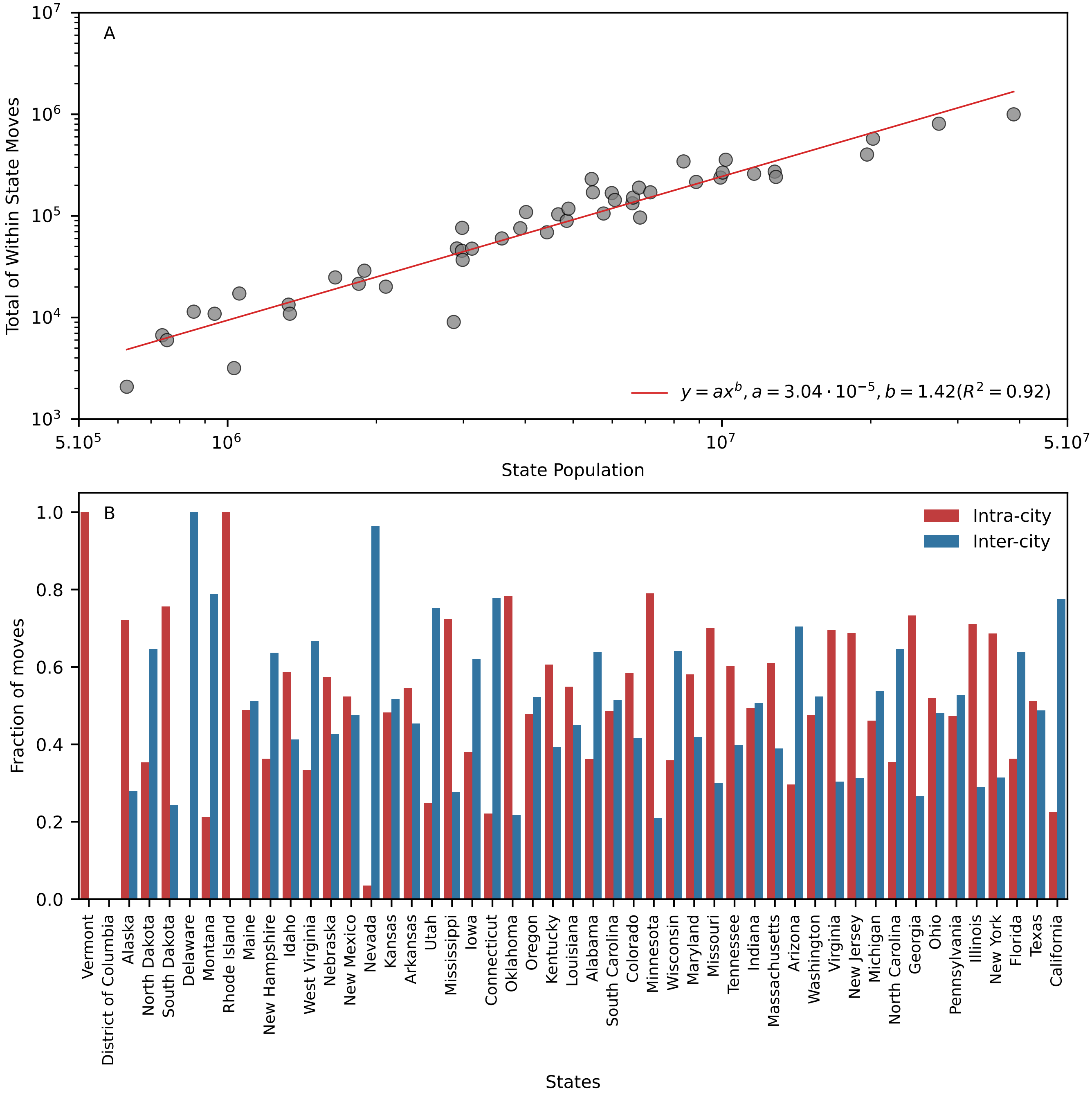

Supplementary Fig. 6: Heterogeneity of within state flows across all states of the U.S.. Flows within state scales linearly with the state population (A). On average, the split between intra- and inter-city flows is equal, but the fraction of within state flows due to intra- and inter-city migration (B) reveals the heterogeneity of within state flows within the U.S..

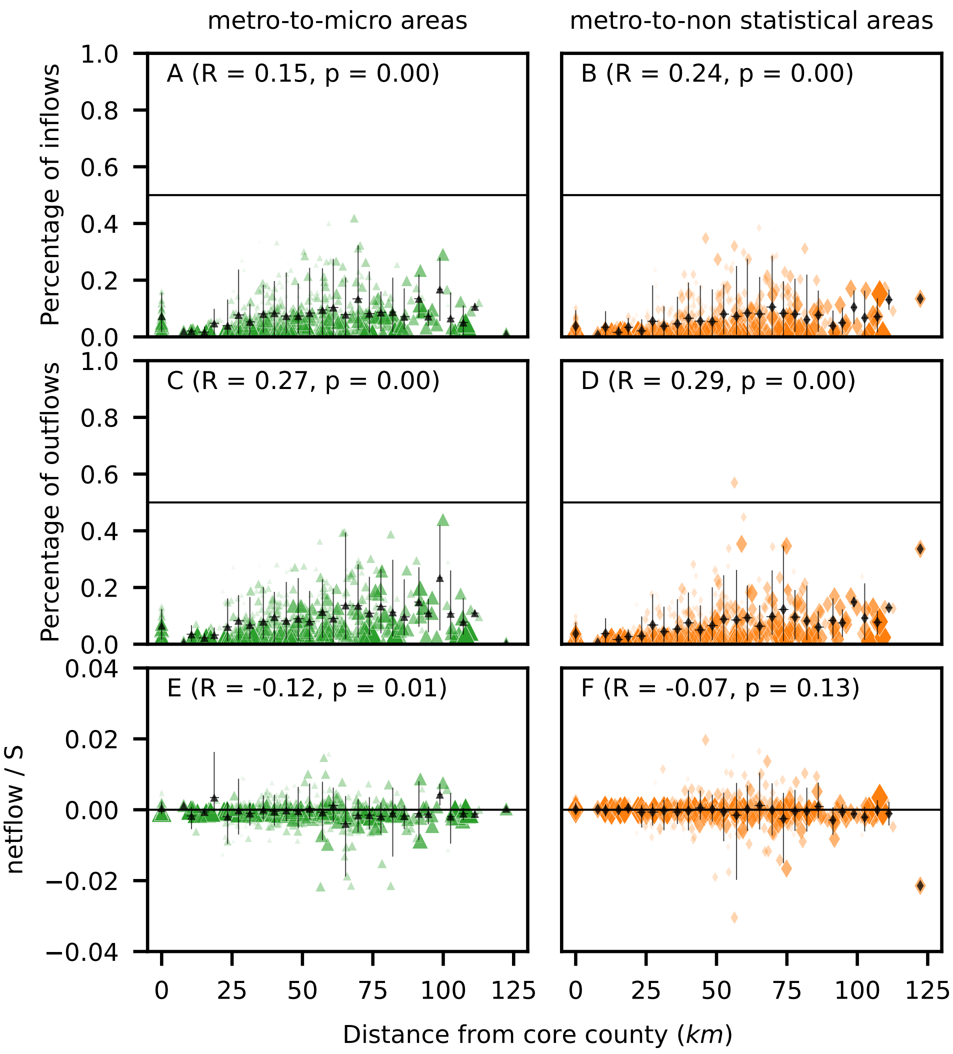

Supplementary Fig. 7: Roles of flows between metro and micro areas, and between metro and non statistical areas in the population growth of cities. The percentage of inflows from micro (A) and from non statistical areas (B) to counties within a city as function their distance to core county. The percentage of outflows to micro and non statistical areas are shown in (C) and (D), respectively. The correlation of the relative growth with distance for metro-to-micro (E) and metro-to-non statistical areas flows in (F) indicate that these flows do not play a significant role in increasing the population of cities. The sizes of green and yellow symbols are proportional to the city population. The range of distances is split into equally spaced bins. The number of counties n within each bin, from left to right, is 46, 1, 4, 7, 7, 17, 21, 31, 36, 38, 34, 31, 31, 30, 20, 20, 21, 14, 17, 9, 9, 6, 4, 2, 5, 5, 2, 1. The black dots and the error bars indicate the mean and the 90% interval, respectively, of the counties within the corresponding bin. We also show the Pearson correlation coefficient R and the p-value associated with the two-sided test of the null hypothesis of non-correlation.

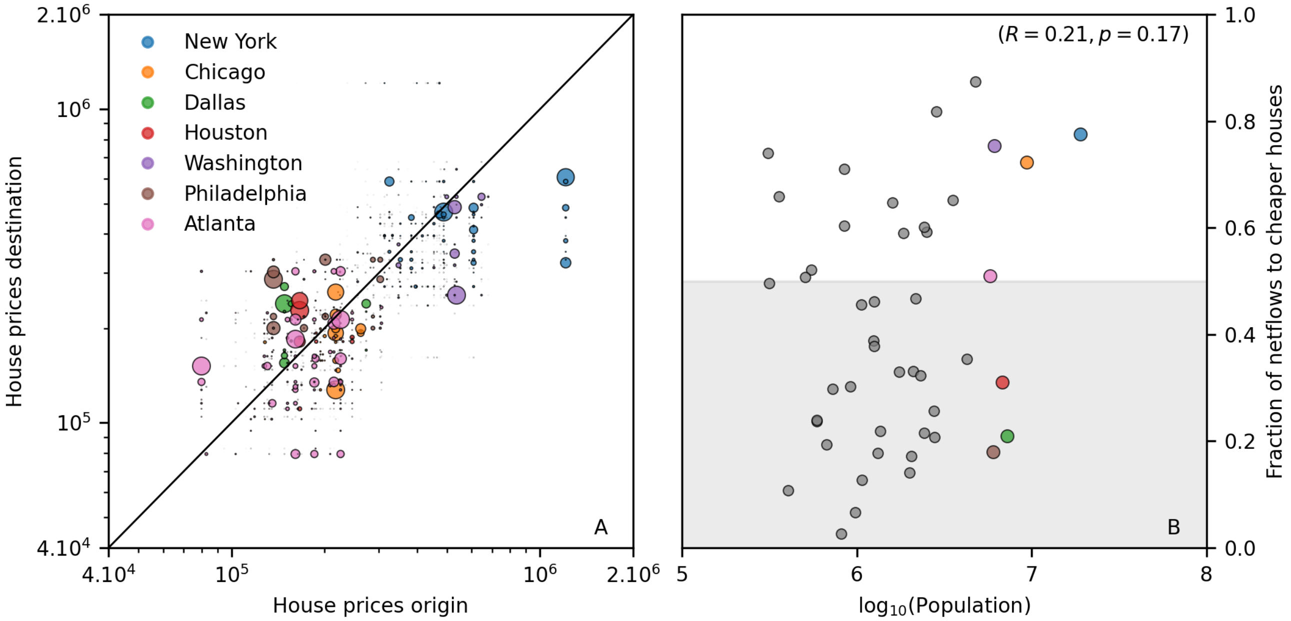

Supplementary Fig. 8: House prices have little to no effect in driving intra-city netflows. Panel A shows the house prices in the origin and destination counties of intra-city netflows for New York, Chicago, Dallas, Houston, Washington D.C., Philadelphia, Atlanta. The size of the symbols are proportional to the intensity of the netflow, and the black line corresponds to y = x. Panel B shows the fraction of netflows to counties with cheaper houses for the 46 MSAs with more than 5 counties. We also show the Pearson correlation coefficient R and the respective p-value associated with the two-sided test of the null hypothesis of non-correlation.

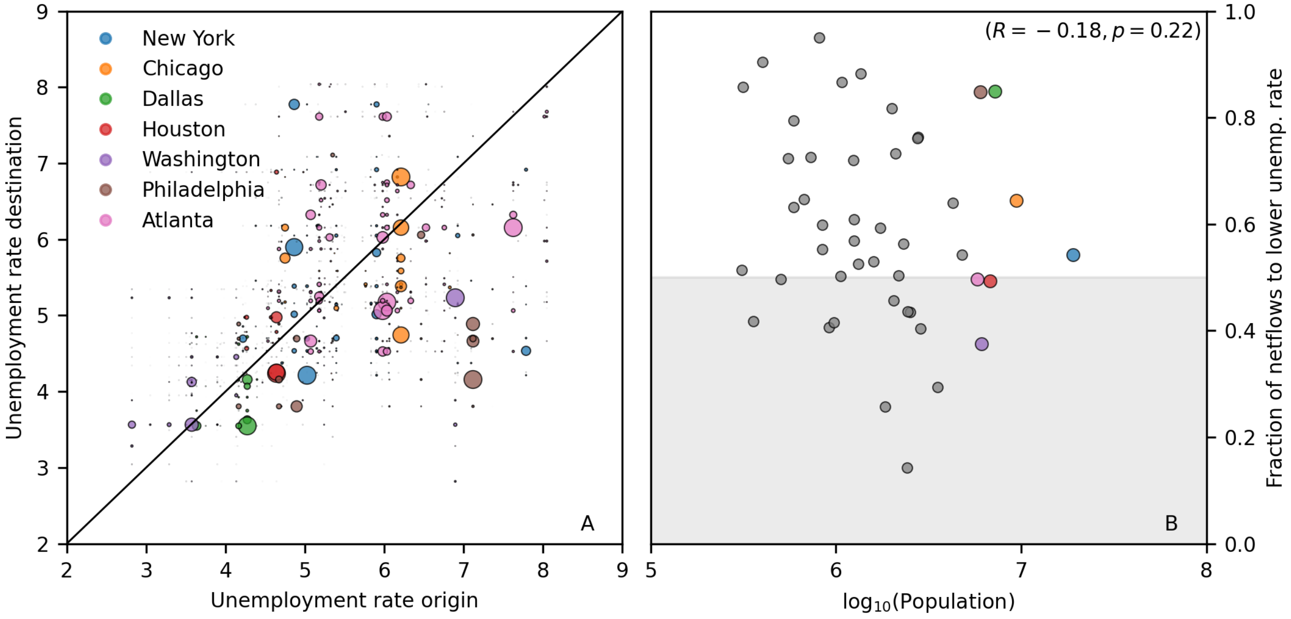

Supplementary Fig. 9: Intra-city netflows of about $2/3$ cities is dominated by moves to counties with lower unemployment rates. Panel A shows the unemployment rate in the origin and destination counties of intra-city netflows for New York, Chicago, Dallas, Houston, Washington D.C., Philadelphia, Atlanta. The size of the symbols are proportional to the intensity of the netflow, and the black line corresponds to y = x. Panel B shows the fraction of netflows to counties with lower unemployment rates for the 46 MSAs with more than 5 counties. We also show the Pearson correlation coefficient R and the respective p-value associated with the two-sided test of the null hypothesis of non-correlation.

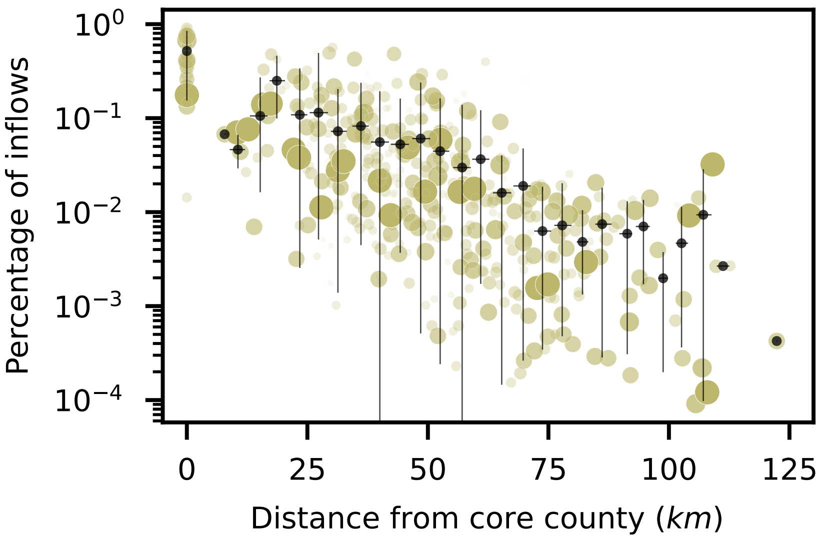

Supplementary Fig. 10: International inflows decay dramatically with the distance from the core county. The highest share of international inflows is concentrated at the core counties of large cities. The range of distances is split into equally spaced bins. The number of counties n within each bin, from left to right, is 46, 1, 4, 7, 7, 17, 21, 31, 36, 38, 34, 31, 31, 30, 20, 20, 21, 14, 17, 9, 9, 6, 4, 2, 5, 5, 2, 1. The black dots and the error bars indicate the mean and the 90% interval, respectively, of the counties within the corresponding bin.

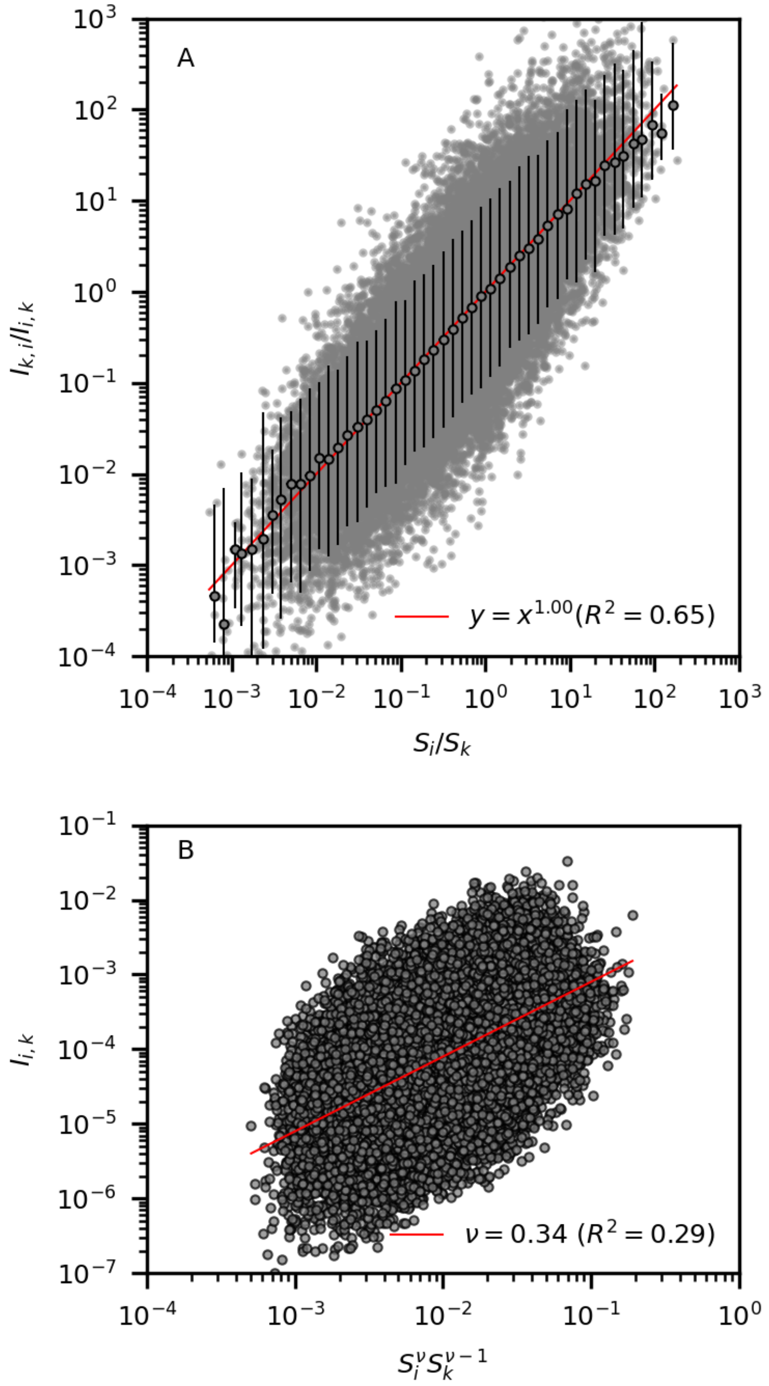

Supplementary Fig. 11: Analysis of inter-city flows between county i and metro area k. We observe a linear relation between $I_{k,i}/I_{i,k}$ and S_i/S_k in (A). The range of the x-axis is split into equally log-spaced bins. The number of samples n within each bin, from left to right, is 5, 11, 7, 15, 23, 48, 65, 95, 155, 214, 320, 423, 512, 724, 917, 1079, 1238, 1456, 1634, 1775, 1871, 2130, 2181, 2327, 2421, 2496, 2408, 2384, 2417, 2297, 2080, 1900, 1647, 1456, 1235, 1073, 774, 592, 439, 343, 235, 174, 136, 76, 50, 30, 16, 5, 3. The black dots and the error bars indicate the mean and the 90% interval, respectively, of the samples within the corresponding bin. In (B), the fitting of $I_{i,k}$ versus $S_i^{\nu} S_k^{\nu-1}$ allows us to obtain the value of the exponent $\nu = 0.34$.

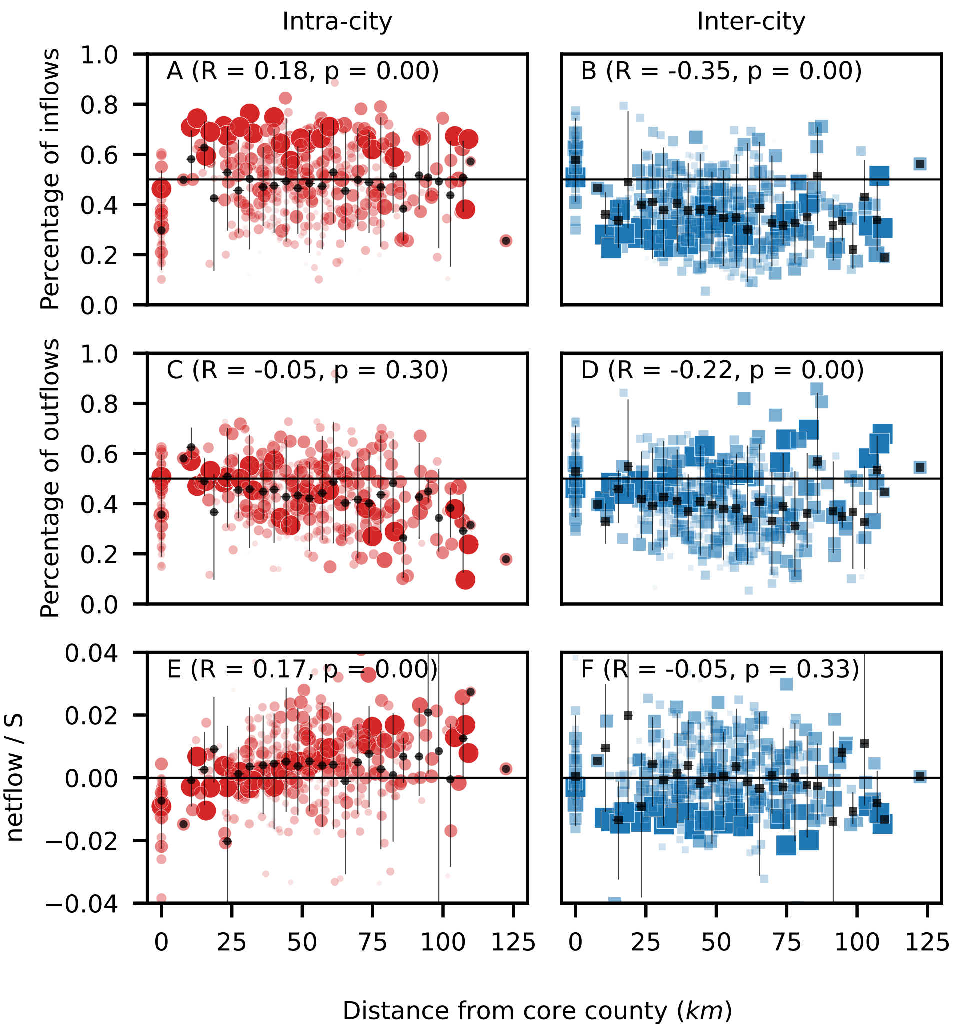

Supplementary Fig. 12: Roles of intra- and inter-city flows in driving the heterogeneous observation of cities for the $2005-2009$ period. The core county is defined as the one with the highest population density, and panels A and B shows the percentage of inflows due to intra- and inter-city flows, respectively. The percentage of outflows due intra- and inter-flows are show in panels C and D. The resulting netflows adjusted by city size due to intra- and inter-city flows are shown in panels E and D. The sizes of red circles and blue squares are proportional to city population of the county. The range of distances is split into equally spaced bins. The number of counties n within each bin, from left to right, is 46, 1, 4, 7, 7, 17, 21, 31, 36, 38, 34, 31, 31, 30, 20, 20, 21, 14, 17, 9, 9, 6, 4, 2, 5, 5, 2, 1. The black dots and the error bars indicate the mean and the 90% confidence interval of the corresponding bin. We also show the Pearson correlation coefficient R and the respective p-value associated with the two-sided test of the null hypothesis of non-correlation.

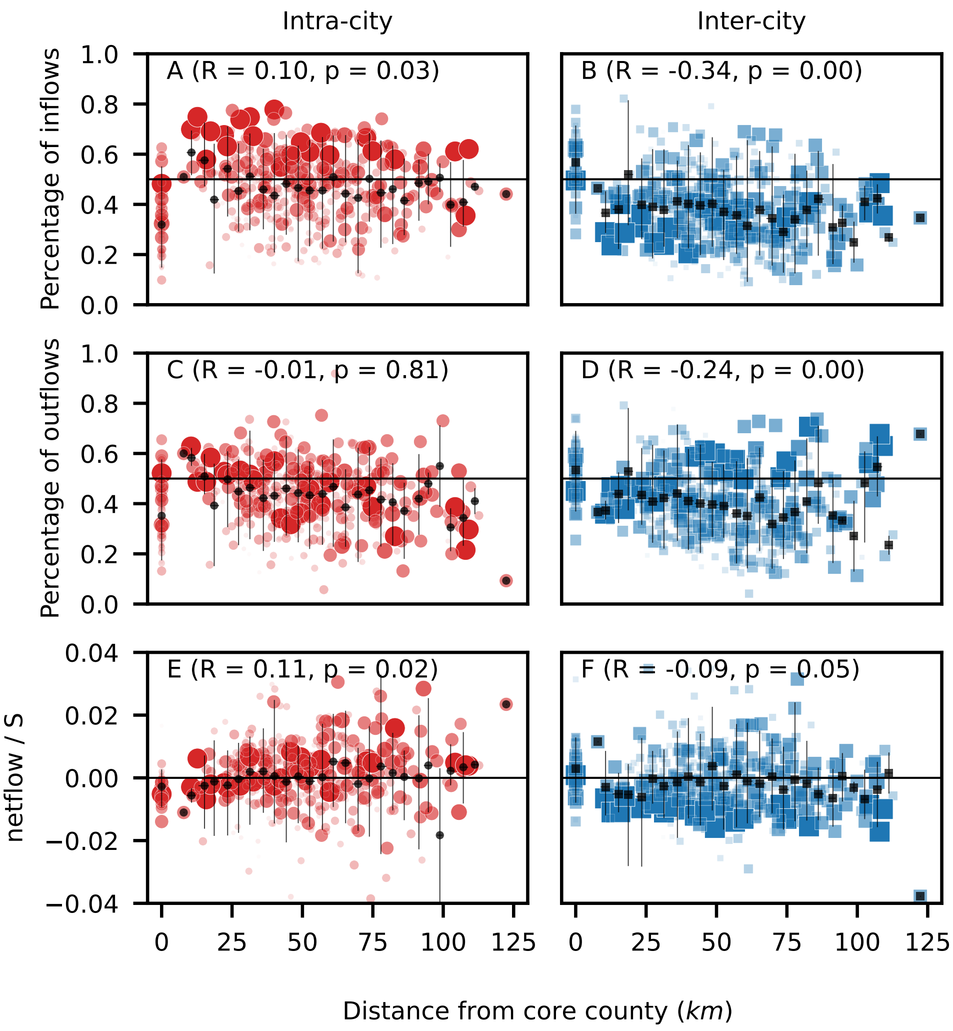

Supplementary Fig. 13: Roles of intra- and inter-city flows in driving the heterogeneous observation of cities for the $2010 - 2014$ period. The core county is defined as the one with the highest population density, and panels A and B shows the percentage of inflows due to intra- and inter-city flows, respectively. The percentage of outflows due intra- and inter-flows are show in panels C and D. The resulting netflows adjusted by city size due to intra- and inter-city flows are shown in panels E and D. The sizes of red circles and blue squares are proportional to city population of the county. The range of distances is split into equally spaced bins. The number of counties n within each bin, from left to right, is 46, 1, 4, 7, 7, 17, 21, 31, 36, 38, 34, 31, 31, 30, 20, 20, 21, 14, 17, 9, 9, 6, 4, 2, 5, 5, 2, 1. The black dots and the error bars indicate the mean and the 90% confidence interval of the corresponding bin. We also show the Pearson correlation coefficient R and the respective p-value associated with the two-sided test of the null hypothesis of non-correlation.

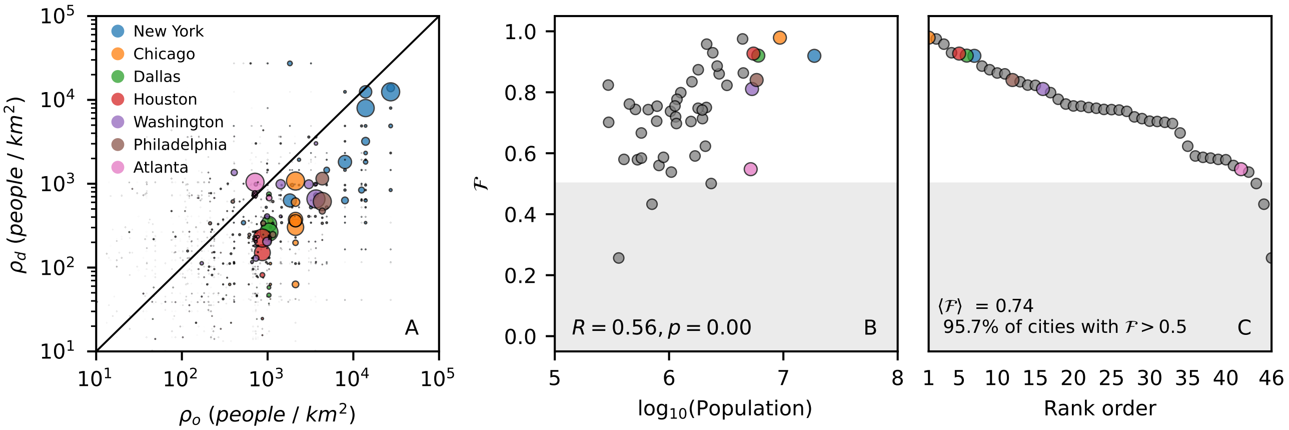

Supplementary Fig. 14: People are moving to counties with lower population density in the period $2005 - 2009$. (A) The population density of the origin (ρ_o) and destination (ρ_d) counties of intra-city netflows for New York, Chicago, Dallas, Houston, Washington D.C., Philadelphia, Atlanta, reveal that the majority of the netflows occur from high to low density counties. The size of the symbols are proportional to the intensity of the netflow, and the black line corresponds to $y = x$. (B) The fraction of netflows to lower density counties $\mathcal{F}$ has a positive correlation with city population when we consider the 46 MSAs with more than 5 counties, suggesting that intra-city flows to lower density counties are more frequent as the city size increases. We also show the Pearson correlation coefficient R and the respective p-value associated with the two-sided test of the null hypothesis of non-correlation. (C) The ranking of the cities according to $\mathcal{F}$.

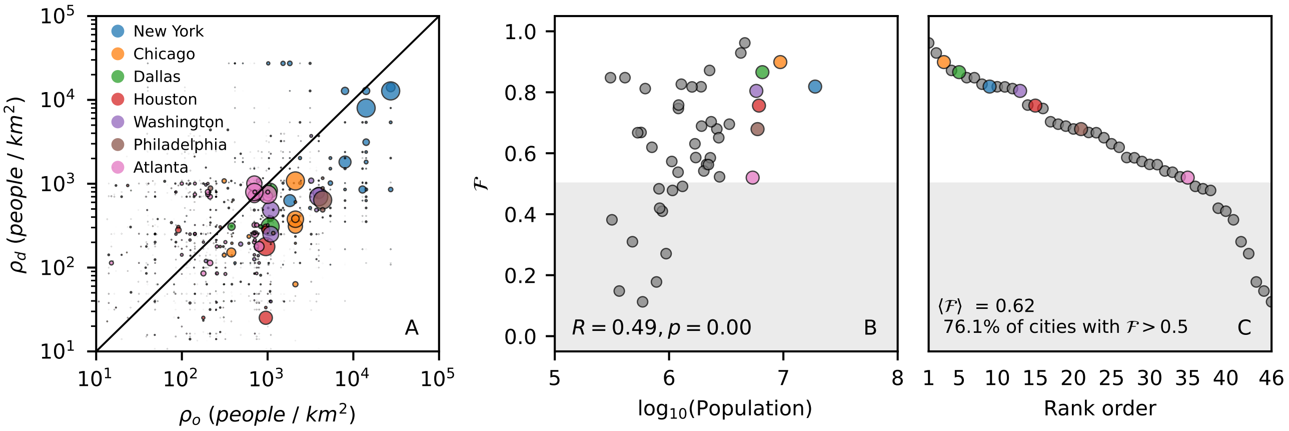

Supplementary Fig. 15: People are moving to counties with lower population density in the period $2010 - 2014$. (A) The population density of the origin (ρ_o) and destination (ρ_d) counties of intra-city netflows for New York, Chicago, Dallas, Houston, Washington D.C., Philadelphia, Atlanta, reveal that the majority of the netflows occur from high to low density counties. The size of the symbols are proportional to the intensity of the netflow, and the black line corresponds to $y = x$. (B) The fraction of netflows to lower density counties $\mathcal{F}$ has a positive correlation with city population when we consider the 46 MSAs with more than 5 counties, suggesting that intra-city flows to lower density counties are more frequent as the city size increases. We also show the Pearson correlation coefficient R and the respective p-value associated with the two-sided test of the null hypothesis of non-correlation. (C) The ranking of the cities according to $\mathcal{F}$.

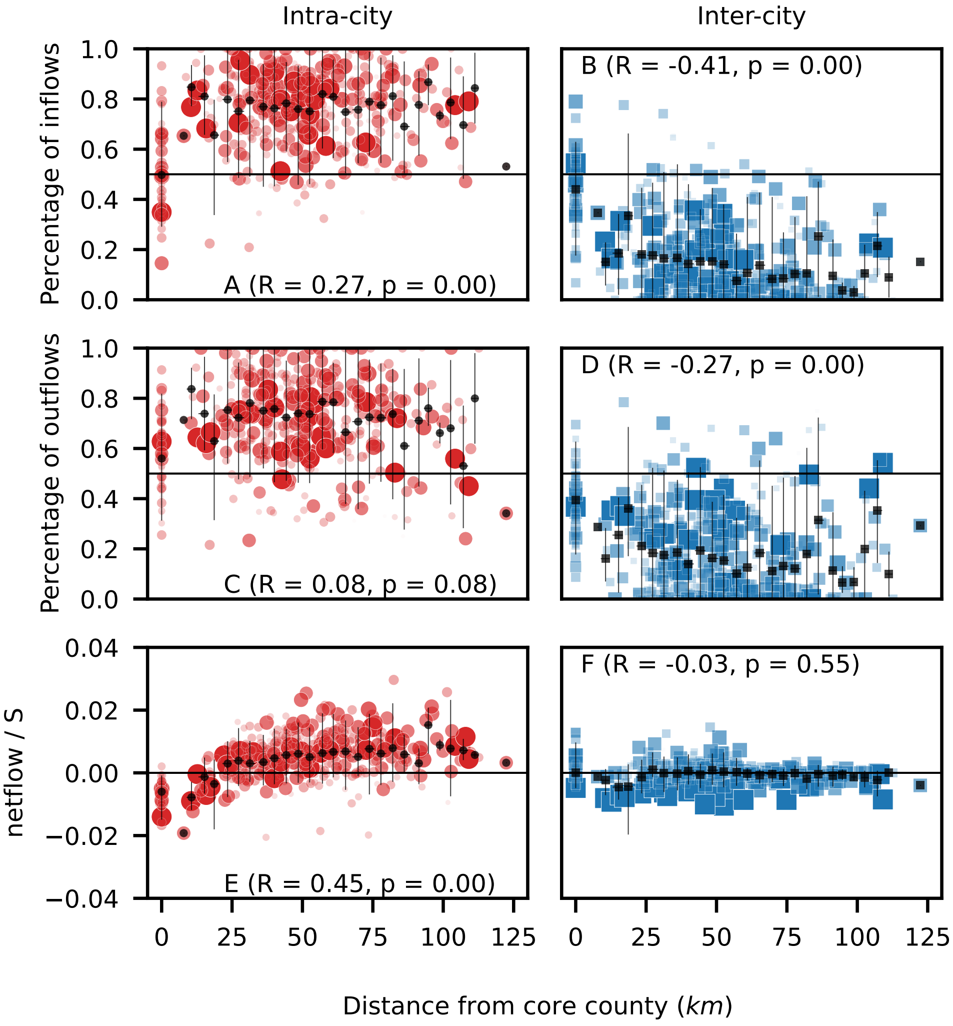

Supplementary Fig. 16: Roles of intra- and inter-city flows in driving the heterogeneous observation of cities for the $2015 - 2016$ period using the IRS dataset. The core county is defined as the one with the highest population density, and panels A and B shows the percentage of inflows due to intra- and inter-city flows, respectively. The percentage of outflows due intra- and inter-city flows are show in panels C and D. The resulting netflows adjusted by city size due to intra- and inter-city flows are shown in panels E and D. The sizes of red circles and blue squares are proportional to city population of the county. The range of distances is split into equally spaced bins. The number of counties n within each bin, from left to right, is 46, 1, 4, 7, 7, 17, 21, 31, 36, 38, 34, 31, 31, 30, 20, 20, 21, 14, 17, 9, 9, 6, 4, 2, 5, 5, 2, 1. The black dots and the error bars indicate the mean and the 90% confidence interval of the corresponding bin. We also show the Pearson correlation coefficient R and the respective p-value associated with the two-sided test of the null hypothesis of non-correlation.

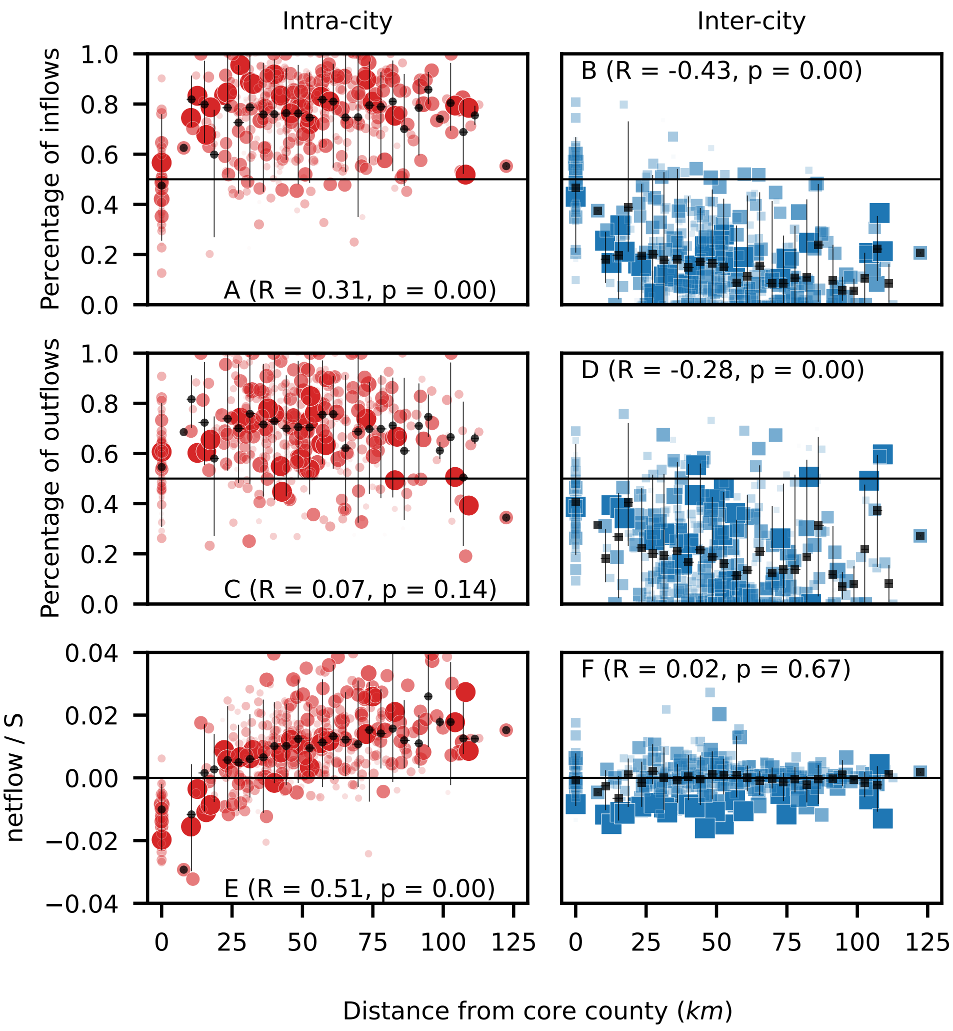

Supplementary Fig. 17: Roles of intra- and inter-city flows in driving the heterogeneous observation of cities for the $2016-2017$ period using the IRS dataset. The core county is defined as the one with the highest population density, and panels A and B shows the percentage of inflows due to intra- and inter-city flows, respectively. The percentage of outflows due intra- and inter-city flows are show in panels C and D. The resulting netflows adjusted by city size due to intra- and inter-city flows are shown in panels E and D. The sizes of red circles and blue squares are proportional to city population of the county. The range of distances is split into equally spaced bins. The number of counties n within each bin, from left to right, is 46, 1, 4, 7, 7, 17, 21, 31, 36, 38, 34, 31, 31, 30, 20, 20, 21, 14, 17, 9, 9, 6, 4, 2, 5, 5, 2, 1. The black dots and the error bars indicate the mean and the 90% confidence interval of the corresponding bin. We also show the Pearson correlation coefficient R and the respective p-value associated with the two-sided test of the null hypothesis of non-correlation.

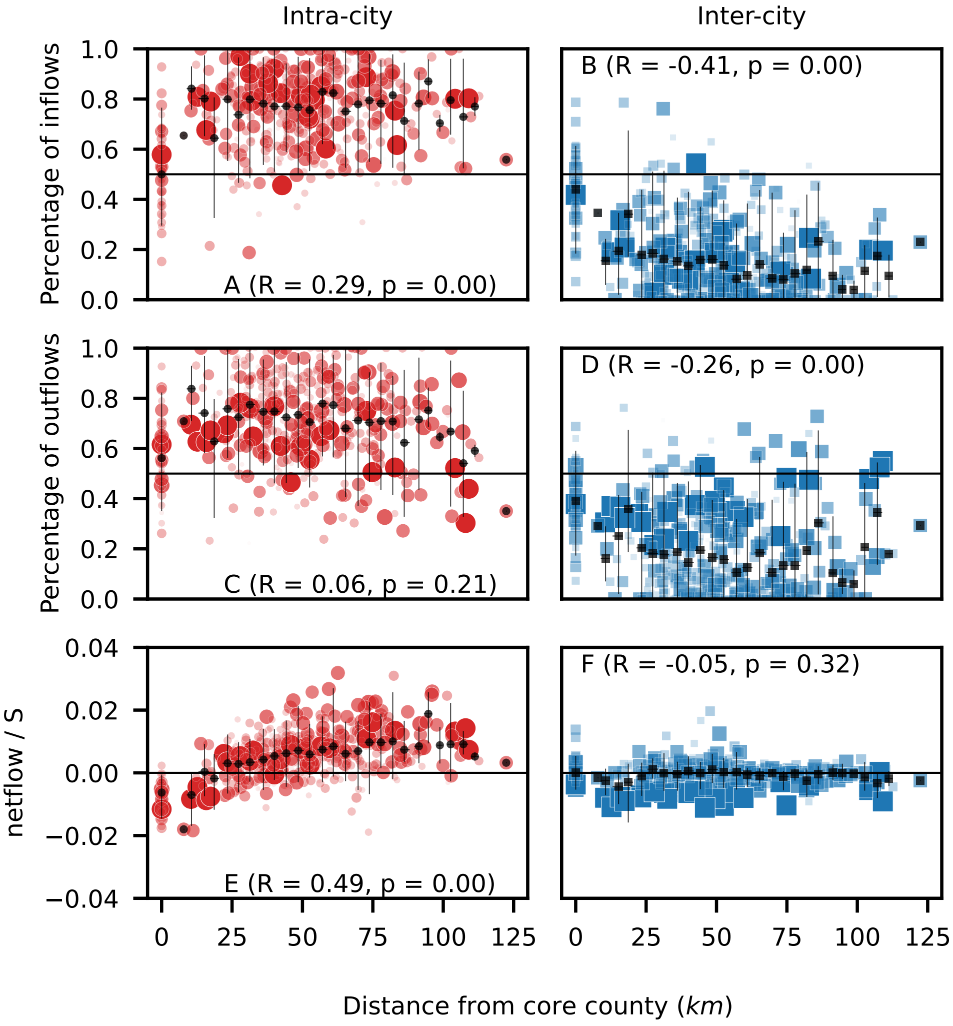

Supplementary Fig. 18: Roles of intra- and inter-city flows in driving the heterogeneous observation of cities for the $2017 - 2018$ period using the IRS dataset. The core county is defined as the one with the highest population density, and panels A and B shows the percentage of inflows due to intra- and inter-city flows, respectively. The percentage of outflows due intra- and inter-city flows are show in panels C and D. The resulting netflows adjusted by city size due to intra- and inter-city flows are shown in panels E and D. The sizes of red circles and blue squares are proportional to city population of the county. The range of distances is split into equally spaced bins. The number of counties n within each bin, from left to right, is 46, 1, 4, 7, 7, 17, 21, 31, 36, 38, 34, 31, 31, 30, 20, 20, 21, 14, 17, 9, 9, 6, 4, 2, 5, 5, 2, 1. The black dots and the error bars indicate the mean and the 90% confidence interval of the corresponding bin. We also show the Pearson correlation coefficient R and the respective p-value associated with the two-sided test of the null hypothesis of non-correlation.

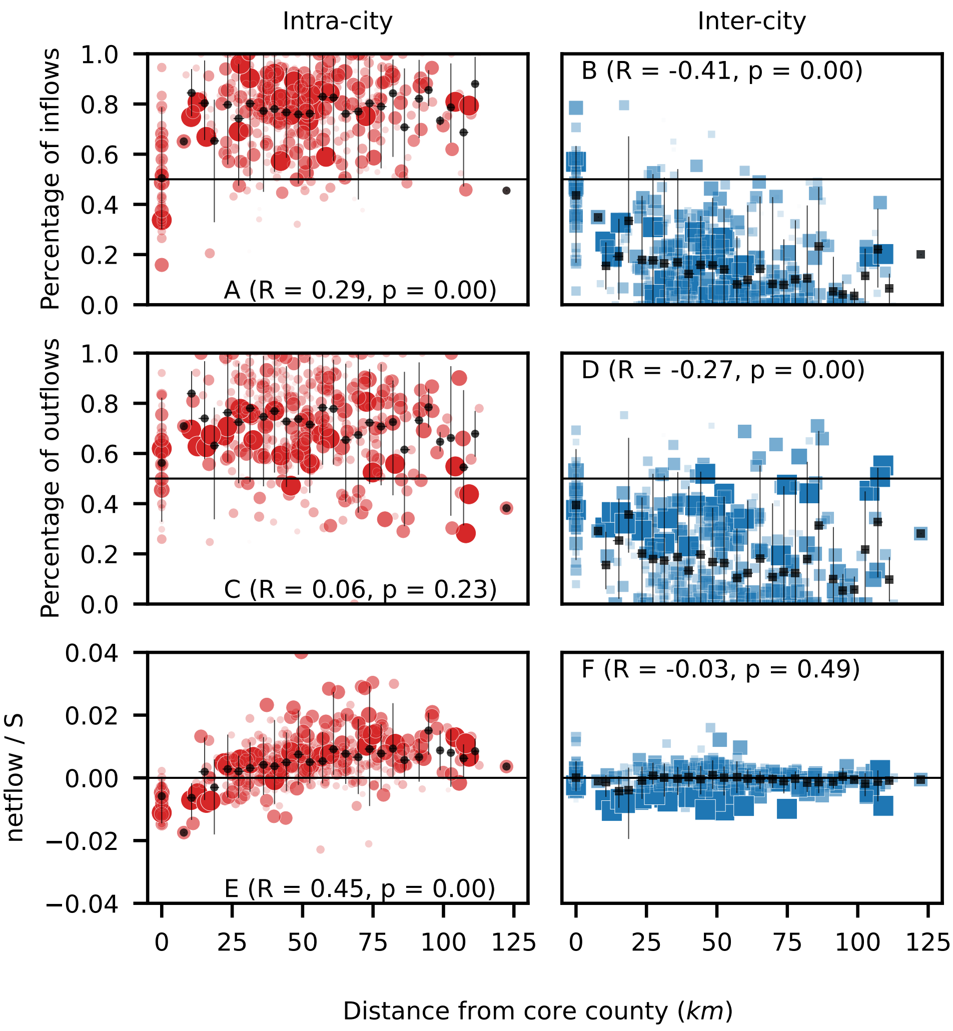

Supplementary Fig. 19: Roles of intra- and inter-city flows in driving the heterogeneous observation of cities for the $2018 - 2019$ period using the IRS dataset. The core county is defined as the one with the highest population density, and panels A and B shows the percentage of inflows due to intra- and inter-city flows, respectively. The percentage of outflows due intra- and inter-city flows are show in panels C and D. The resulting netflows adjusted by city size due to intra- and inter-city flows are shown in panels E and D. The sizes of red circles and blue squares are proportional to city population of the county. The range of distances is split into equally spaced bins. The number of counties n within each bin, from left to right, is 46, 1, 4, 7, 7, 17, 21, 31, 36, 38, 34, 31, 31, 30, 20, 20, 21, 14, 17, 9, 9, 6, 4, 2, 5, 5, 2, 1. The black dots and the error bars indicate the mean and the 90% confidence interval of the corresponding bin. We also show the Pearson correlation coefficient R and the respective p-value associated with the two-sided test of the null hypothesis of non-correlation.
