# Supplementary material for: Spatial structure of city population growth
Source: Nat Commun. 2022 Oct 8;13:5931. doi: 10.1038/s41467-022-33527-y (PMC9547901; doi:10.1038/s41467-022-33527-y)
Supplement: Supplementary file 2 — Reporting Summary [file 41467_2022_33527_MOESM2_ESM.pdf]

nature portfolio

Corresponding author(s): Dr Satish Ukkusuri

Last updated by author(s): Sep 16, 2022

Reporting Summary

Nature Portfolio wishes to improve the reproducibility of the work that we publish. This form provides structure for consistency and transparency in reporting. For further information on Nature Portfolio policies, see our Editorial Policies and the Editorial Policy Checklist.

Statistics

For all statistical analyses, confirm that the following items are present in the figure legend, table legend, main text, or Methods section.

n/a	Confirmed	
☐	☒	The exact sample size (n) for each experimental group/condition, given as a discrete number and unit of measurement
☐	☒	A statement on whether measurements were taken from distinct samples or whether the same sample was measured repeatedly
☐	☒	The statistical test(s) used AND whether they are one- or two-sided<br>*Only common tests should be described solely by name; describe more complex techniques in the Methods section.*
☒	☐	A description of all covariates tested
☒	☐	A description of any assumptions or corrections, such as tests of normality and adjustment for multiple comparisons
☐	☒	A full description of the statistical parameters including central tendency (e.g. means) or other basic estimates (e.g. regression coefficient) AND variation (e.g. standard deviation) or associated estimates of uncertainty (e.g. confidence intervals)
☐	☒	For null hypothesis testing, the test statistic (e.g. F, t, r) with confidence intervals, effect sizes, degrees of freedom and P value noted<br>*Give P values as exact values whenever suitable.*
☒	☐	For Bayesian analysis, information on the choice of priors and Markov chain Monte Carlo settings
☒	☐	For hierarchical and complex designs, identification of the appropriate level for tests and full reporting of outcomes
☐	☒	Estimates of effect sizes (e.g. Cohen's d, Pearson's r), indicating how they were calculated

Our web collection on statistics for biologists contains articles on many of the points above.

Software and code

Policy information about availability of computer code

Data collection	We downloaded data from the U.S. Census , IRS and Zillow websites and loaded them onto python-pandas DataFrames.
Data analysis	The data were analyzed in Python 3.7.12, using the following libraries: pandas 1.3.4, matplotlib 3.5.1, numpy 1.18.5, statsmodels 0.13.2, sklearn 1.0.2, scipy 1.7.3. The codes to create the figures will be made available upon request.

For manuscripts utilizing custom algorithms or software that are central to the research but not yet described in published literature, software must be made available to editors and reviewers. We strongly encourage code deposition in a community repository (e.g. GitHub). See the Nature Portfolio guidelines for submitting code & software for further information.

Data

Policy information about availability of data

All manuscripts must include a data availability statement. This statement should provide the following information, where applicable:

- Accession codes, unique identifiers, or web links for publicly available datasets
- A description of any restrictions on data availability
- For clinical datasets or third party data, please ensure that the statement adheres to our policy

This paper uses datasets from the following publicly available sources:

County-to-county migration flow files: https://www.census.gov/data/tables/2019/demo/geographic-mobility/county-to-county-migration-2015-2019.html

County population totals: https://www.census.gov/data/datasets/time-series/demo/popest/2010s-counties-total.html

IRS Migration data: https://www.irs.gov/statistics/soi-tax-stats-migration-data

Zillow Research: https://www.zillow.com/research/data/

Human research participants

Policy information about studies involving human research participants and Sex and Gender in Research.

Reporting on sex and gender	n/a
Population characteristics	n/a
Recruitment	n/a
Ethics oversight	n/a

Note that full information on the approval of the study protocol must also be provided in the manuscript.

Field-specific reporting

Please select the one below that is the best fit for your research. If you are not sure, read the appropriate sections before making your selection.

☐ Life sciences ☐ Behavioural & social sciences ☒ Ecological, evolutionary & environmental sciences

For a reference copy of the document with all sections, see nature.com/documents/nr-reporting-summary-flat.pdf

Ecological, evolutionary & environmental sciences study design

All studies must disclose on these points even when the disclosure is negative.

Study description

We use county-to-county flow data to analyze the domestic migration pattern of U.S. cities.

Research sample

We have used ACS 5-Year Migration Flow Files to analyze migration flows between counties within the United States. Such datasets are suitable for our purposes because we can identify the origin and destination counties of population flows. These datasets are representative since they include migration flows to and from all the counties within the United States.

Sampling strategy

In this paper, we study the effects of domestic netflows on the spatial heterogeneity of population growth. Given that the U.S. is a highly urbanized country, our sample is composed of counties belonging to metropolitan statistical areas.

Data collection

We used two datasurces from the U.S. Census.
The first source from Census is the ACS County-to-County Migration Files.
There were four modes of data collection: internet, mail, telephone, and personal visit, in which respondents are asked whether they lived in the same residence one year ago.

The second source from Census is the County Population Totals: 2010-2019 dataset, which offers components of population change from April 1, 2010 to July 1, 2019.
Using the resident population from the 2010 Census as a starting point (population base), county population estimates are derived from a demographic balancing equation

To analyze the housing prices of origin and destination counties, we used the housing data from Zillow Research.
Zillow publishes the Zillow Home Value Index, which reflects the typical values of homes across the U.S..

The IRS data we used to check the robustness of our findings were collected from the IRS - SOI Tax Stats - Migration Data website, which reports migration data in the U.S. based on year-to-year address changes reported on individual income tax returns filed with the IRS.

Timing and spatial scale

Our main analysis is composed of population flows collected within the 2015-2019 period in the United States. The robustness of our findings are verified by extending our analysis to other time periods as well, ranging from 2005 to 2014. The data we used were collected at the county level.

Data exclusions

The ACS migration flow files supply population flows to and from all the counties within the United States. Inflows and outflows between counties belonging to metropolitan statistical areas are analyzed in details in the main manuscript, while population flows between metro and rural counties are briefly addressed at the Supplementary Information. International inflows are also reported in the manuscript. We excluded from our analysis population flows between rural areas because: (1) the highest share (86%) of the U.S. population lives in metropolitan areas; (2) flows between rural areas do not affect the spatial heterogeneity of city growth.

Reproducibility	The consistency of our county-level findings were addressed by aggregating the data at the city level and reproducing published results. Apart from that, our findings for 2015-2019 period were verified by extending our analysis to other non-overlapping periods, 2005-2009 and 2010-2014, and by extending our analysis to the same period but using other datasets (IRS).
Randomization	Our study is focused on the analysis of population flows from US census. Our goal is to examine general patterns of migration intra- and inter-cities, and to show the spatial heterogeneity of city population growth. In this context, randomization is not relevant to our study because the spatial heterogeneity of the counties is the key aspect of our analysis.
Blinding	This is not relevant to our study because we do not collect data from individuals: our analysis is perfomed on population data made available by Census and IRS.

Did the study involve field work? ☐ Yes ☒ No

Reporting for specific materials, systems and methods

We require information from authors about some types of materials, experimental systems and methods used in many studies. Here, indicate whether each material, system or method listed is relevant to your study. If you are not sure if a list item applies to your research, read the appropriate section before selecting a response.

Materials & experimental systems

n/a	Involved in the study
☒	☐ Antibodies
☒	☐ Eukaryotic cell lines
☒	☐ Palaeontology and archaeology
☒	☐ Animals and other organisms
☒	☐ Clinical data
☒	☐ Dual use research of concern

Methods

n/a	Involved in the study
☒	☐ ChIP-seq
☒	☐ Flow cytometry
☒	☐ MRI-based neuroimaging
